# Pulmonary sequestration presented as massive left hemothorax and associated with primary lung sarcoma

**DOI:** 10.1186/1471-2482-13-S2-S34

**Published:** 2013-10-08

**Authors:** Vincenzo Di Crescenzo, Paolo Laperuta, Filomena Napolitano, Chiara Carlomagno, Alfredo Garzi, Mario Vitale

**Affiliations:** 1 Department of Medicine and Surgery, University of Salerno, Italy; 2 Department of Clinical Medicine and Surgery, University of Naples "Federico II", Italy

**Keywords:** Pulmonary Sequestration, hemithorax, primary sarcoma

## Abstract

Pulmonary sequestration is an uncommon disease, accounting for only approximately 1.5% of all congenital pulmonary malformations. In most cases, the diagnosis is a result of accidental radiological findings; it is rarely accompanied by clinical symptoms, and is more commonly associated with other congenital malformations. Herein, we reported a case of pulmonary sequestration presented as massive left hemothorax and associated with primary lung sarcoma. A pneumonectomy via thoracotomy was attended with complete resection of sequestration and of sarcoma. The postoperative course was unremarkable, and the patient was discharged on postoperative day 11.

## Backgroud

Pulmonary sequestration is an uncommon disease, accounting for only approximately 1.5% of all congenital pulmonary malformations [1-3]. In most cases, the diagnosis is a result of accidental radiological findings; it is rarely accompanied by clinical symptoms, and is more commonly associated with other congenital malformations. In the present case pulmonary sequestration presented as massive left hemothorax was associated with primary lung sarcoma. A pneumonectomy via thoracotomy was attended with complete resection of sequestration and of sarcoma.

## Case presentation

**Figure 1:**
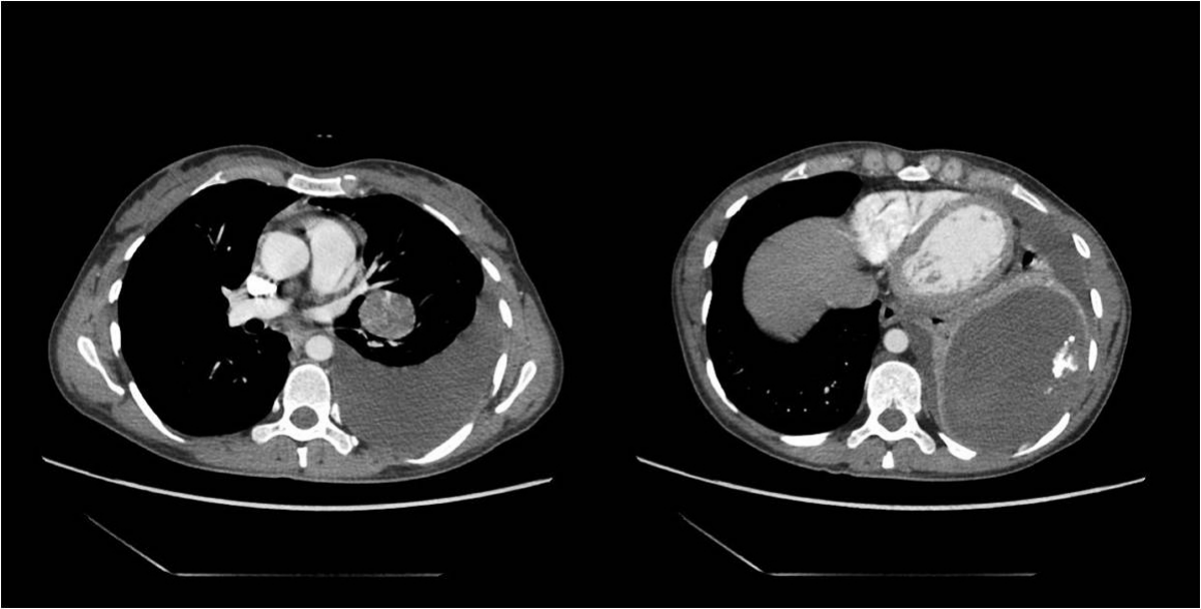


**Figure 2:**
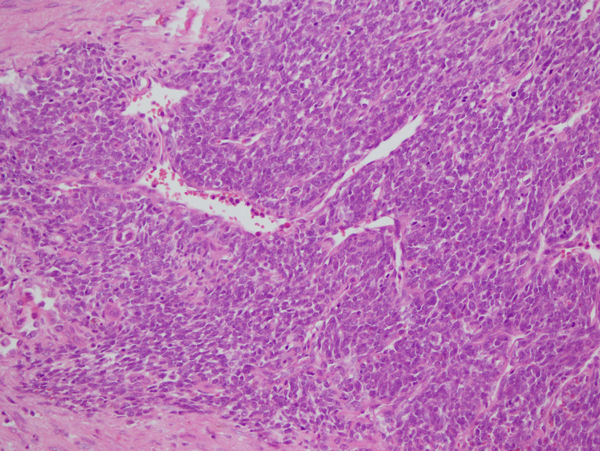
**Histological features of the primary pulmonary fibrosarcoma showing a momorphous population of small and undifferentiated spindle cells (Haematoxylin Eosin 430X)**.

A 35 years-old woman was admitted to local hospital complained of dyspnea, cough and hemoptosis. A Chest X-ray showed the presence of massive left pleural effusion. Thus, she referred to our unit for the treatment. A chest computed tomography (CT) scan confirmed the presence of pleural effusion accompanied by an area of consolidation. Pleural puncture undoubtedly revealed hemothorax. A tube thoracostomy yielded hemothorax to the amount of 1100 ml. The clinical condition of patients was stable and all laboratory values were within normal. A repeated CT scan performed three days after the procedure, confirmed the presence of area of consolidation in lower left lobe and the presence of an hilar nodule (size 3 mm) not been reported before (Figure 1). Bronchoscopy showed no endoluminal lesion and the pathological results from bronchoalveolar lavage showed the presence of inflammatory cells in absence of malignant cells [4,5]. Then a fine needle biopsy CT-guided was attended but the results was inconclusive for a definitive diagnosis. Arteriography diagnosed the mass located in lower lobe to be a sequestration. Thus, our strategy was to attend an exploratory thoracotomy. The sequestration was successfully resected with partial resection of the diaphragm. Then the nodule was biopsied and the intraoperative diagnosis was positive for malignancy, possibly a sarcoma, whereas a conclusive diagnosis on frozen sections specimen was not possible. The tumor invaded the main trunk of left pulmonary artery, thus contraindicated a bronchoplastic procedure. A pneumonectomy was attended in a standard manner. A chest drainage was left in the chest, and removed two days after. For postoperative pain control, a patient controlled analgesia was used for the first two days [6]. Pathologic examinations revealed the sequestration to be an infarcted extralobar sequestration (ELS). Small elastic arteries were identified within the sequestration. The final diagnosis of the tumor was primary fibrosarcoma of the lung according to the pathological and immunostaining results as reported in Figures 2. And 3. The postoperative course was unremarkable, and the patient was discharged on postoperative day 11.

**Figure 3:**
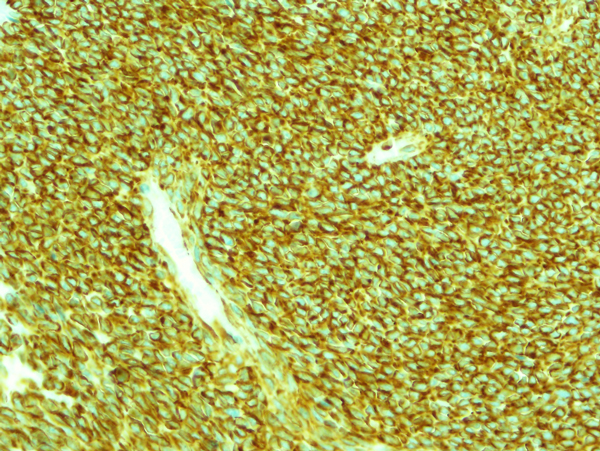
**Immunohistochemical features of the primary pulmonary fibrosarcoma showing diffuse positivity for Vimentin (APAAP 430X)**.

## Discussion

Pulmonary sequestration is defined as a mass of nonfunctioning lung tissue that receives blood supply from an anomalous systemic artery and does not communicate with the normal bronchial tree, being divided into two forms: intralobar sequestration (ILS) and extralobar sequestration (ELS) [1-3] Whereas ELS is enclosed within its own pleural membrane, ILS shares the pleural membrane of the normal lung. Considered a congenital disease, ELS results from an accessory lung bud that, in some cases, maintains the original connection with the intestine, allowing communication between the sequestration and the gastrointestinal tract. The arterial blood supply to the sequestered lung by aberrant elastic arteries mostly originates from the thoracic or abdominal aorta in most cases with EPS. These arteries may arise above or below the diaphragm, and are usually small and variable [1]. The left hemithorax is the most commonly affected (in 65% of cases), and the primary site of ELS is the region between the lower lung lobe and the diaphragm (in 63%), as in the present case. In more than 60% of all cases of ELS, other congenital anomalies coexist, the most common of which is diaphragmatic hernia (in 16%). In approximately 25% of the cases, another pulmonary abnormality, such as pulmonary hypoplasia, cystic adenomatoid malformation or congenital lobar emphysema, is also present [2]. The treatment for pulmonary sequestration is surgery. In the case of ELS, sequestrectomy should be performed. The identification and control of the aberrant artery branch, above or below the diaphragm, are essential for preventing hemorrhage. Postoperative results are typically excellent [3].

Among the previous reports detected by PubMed using key words for symptomatic EPS such as hemothorax, infarction or torsion, only few cases with symptomatic EPS were cited. The symptoms of these cases were ipsilateral chest pain and respiratory failure with sudden onset caused by hemothorax and infarction. The location of the EPS was the left costovertebral angle adjacent to the inferior ligament in all patients. Based on these previous reports and our experience, symptomatic EPS may be related to infarction caused by torsion under exposure to drastic pleural cavity movement or by infection associated with bacteremia [2]. Conversely, we did not find in literature any paper in which the concomitant presence of pulmonary sequestration and primary lung sarcoma was described. Primary sarcoma of the lung is a rare tumor [7]. In general, sarcomas disseminate by the hematogenous route; consequently, metastases are found frequently in the lungs. Therefore, in any case of pulmonary sarcoma, a total body survey is necessary to exclude a primary tumor elsewhere. That because primary pulmonary sarcomas must be distinguished from the more frequent occurrence of sarcoma metastatic to the lung, primary pulmonary sarcomatoid carcinoma, and diffuse malignant mesothelioma involving the lung. Diagnosis of primary pulmonary sarcoma is not easy without thoracotomy. There are no specific presenting symptoms, and roentgenologic manifestations were not characteristic. It is difficult, if not impossible, to get a correct diagnosis of a malignant mesenchymal tumor from cytologic examination of a (needle) aspirate. Tumor biopsy is necessary to get a diagnosis, and tumor tissue was obtained only before thoracotomy in case of endoluminal growth of a tumor. During the operation, the malignant mesenchymal nature of the tumor may not become clear on macroscopic appearance [8]. It may be helpful to take a biopsy specimen for histologic examination of a frozen section specimen. Once the diagnose has been established (during operation) radical resection appears to be the only treatment with good prognosis. Sometimes, the final diagnosis is obtained postoperative after complete resection of the tumor as in the present case. That because the availability of multiple cytokeratin antibody tests has improved the identification of sarcomatoid carcinomas and diffuse malignant mesothelioma. The introduction of CD34 has facilitated the recognition of both benign and malignant intrathoracic, fibrous tumors [9-16]. Finally, we reported an exceptional case of ELS associated with primary sarcoma of the lung, not been reported in literature before. Both lesions were successfully removed with a left penumonectomy. In case of hemothorax and the presence of multiple lesions (as the present case), a surgical exploration via thoracotomy is required in order to remove all lesions and obtain a definitive diagnosis.

## Competing interests

The authors declare that they have no competing interests.

## Declarations

Funding for publication of the article has come from the: POMPEANGAS Via S. Sebastiano n. 16, ANGRI (SA) Italia. P.IVA 02600816055.

This article has been published as part of *BMC Surgery *Volume 13 Supplement 2, 2013: Proceedings from the 26th National Congress of the Italian Society of Geriatric Surgery. The full contents of the supplement are available online at http://www.biomedcentral.com/bmcsurg/supplements/13/S2
